# MSC-Derived Exosomes can Enhance the Angiogenesis of Human Brain MECs and Show Therapeutic Potential in a Mouse Model of Parkinson's Disease

**DOI:** 10.14336/AD.2020.1221

**Published:** 2021-08-01

**Authors:** Chunling Xue, Xuechun Li, Li Ba, Mingjia Zhang, Ying Yang, Yang Gao, Zhao Sun, Qin Han, Robert Chunhua Zhao

**Affiliations:** ^1^Institute of Basic Medical Sciences of the Chinese Academy of Medical Sciences, School of Basic Medicine, Peking Union Medical College, Peking Union Medical College Hospital, Center of Excellence in Tissue Engineering of Chinese Academy of Medical Sciences, Beijing Key Laboratory, Beijing, China.; ^2^Department of oncology, Peking Union Medical College Hospital, Chinese Academy of Medical Science and Peking Union Medical College, Beijing, China.

**Keywords:** MSCs, exosomes, angiogenesis, PD, SMAD3 and P38MAPK signaling pathways

## Abstract

Parkinson’s disease (PD) is the second most widespread neurodegenerative disorder in the world. It has been reported that exosomes derived from mesenchymal stem cells (MSCs) can contribute to the recovery of PD. However, the underlying mechanism remains poorly defined. In this study, proteomics and time-series analysis showed that exosomes derived from MSCs can keep human brain microvascular endothelial cells (HBMECs) in a transcriptionally active state, which may be beneficial for angiogenesis. Next, we found that MSC-derived exosomes can promote the angiogenesis of HBMECs by increasing the expression of ICAM1, and alleviate the damage caused by 1-methyl-4-phenylpyridinium (MPP+) in these cells. Accordingly, when ICAM1 was knocked down, the tube formation ability of HBMECs was obviously decreased. In addition, ICAM1 was found to promote the angiogenesis of HBMECs by activating the SMAD3 and P38MAPK signaling pathways. In a PD mouse model, MSC-derived exosomes were found to contribute to the recovery of PD by promoting ICAM1-related angiogenesis. These findings demonstrate that the exosome-ICAM1-SMAD3/P38MAPK axis can promote the angiogenesis of HBMECs, with possible therapeutic potential for PD.

Parkinson's disease (PD) is a common progressive neurodegenerative disorder characterized by tremors and delayed movement [1]. The underlying pathological features include the progressive degeneration of dopaminergic pathways in the substantia nigra and striatum, the loss of neurons, and depletion of dopamine (DA) [2]. In addition, surviving dopaminergic neurons of PD patients contain Lewy bodies, which are composed of insoluble aggregated α-synuclein (α-syn) [3, 4]. Neurons and vascular cells constitute the neurovascular unit (NVU), a functionally integrated network that can secrete growth factors and adhesion molecules, which not only regulate the survival of neurons, but also maintain vascular homeostasis and enhance angiogenesis [5-7]. Accordingly, studies have shown that VEGF may mediate angiogenesis and improve neuron survival [8, 9]. Disorders of the NVU, especially those causing unusual neuronal-vascular relationships, play a critical role in the progression of neurodegenerative diseases, including PD [10-13]. Conversely, there is evidence that improvement of NVUs is correlated with PD recovery [14]. Based on these findings, we wanted to investigate whether specifically inducing the improvement of NVUs can promote PD recovery, which may be an effective mechanism for treatment. Clinical trials have indicated that stem cell repair or replacement are promising therapeutic approaches for PD, for which it is imperative to find innovative therapeutic methods to replace damaged neurons [15]. Mesenchymal stromal cells (MSCs) act as multipotent cells, offering great promise for the therapy of various neurological diseases such as PD. Their regenerative effect is thought to mainly rely on the secretion of growth factors and exosomes, or reducing neuroinflammation [16]. There is increasing evidence that extracellular vesicles play a critical role in intercellular communication through the release of proteins, lipids and nucleic acids [17-21]. Exosomes derived from stem cells not only regulate normal physiological processes such as tissue repair [22] and immune surveillance [23, 24], but also contribute to pathological processes such as kidney injury [22] and autoimmune connective tissue diseases [25].

The aim of this study was to evaluate whether exosomes derived from MSCs may contribute to angiogenesis, which in turn can promote the recovery of PD.

## MATERIALS AND METHODS

### Cell culture

The extraction and culture of MSCs was conducted as described in a previous report [26]. HBMECs were purchased from Beina Chuanglian Biotechnology Institute (Beijing, China).

### Extraction and characterization of exosomes

The MSC-derived exosomes were extracted as described previously [27]. For characterization, the purified exosomes were fixed with 1% glutaraldehyde in PBS (pH 7.4) for 0.5 h at room temperature and washed with water. Then, 20 µL of the resulting suspension was loaded onto a Formvar carbon-coated grid, stained with 3% (w/v) aqueous phosphotungstic acid for 1 min at room temperature, and observed by transmission electron microscopy.

### Uptake of exosomes

Exosomes uptake was observed by labeling exosomes with 1'-dioctadecyl-3,3,3',3-tetramethylindocarbocyanine perchlorate (DiL) (Invitrogen). Cell nuclei were counter-stained with Hochest33342. Transfer of the dye was observed by fluorescence microscopy at a final exosome concentration of 200?μg/mL.

### Proteomics

The samples were divided into three groups, including HBMECs, MPTP-treated HBMECs and exosome/MPTP-treated HBMECs. The specific protocols and analyses were performed by Lu-Ming Biotech (China).

### Matrigel tube experiment

For in-vitro tube formation, we added 200 µL of Matrigel (Sigma-Aldrich) to precooled wells of a 24-well plate and incubated it at 37 °C for 15 min. HBMECs were seeded at a density of 1×10^5^/well, and tube formation was observed using an inverted microscope and photographed at different time points (0, 0.5, 2, 6, 12 h).

For in-vivo tube formation, HBMECs were collected and resuspended at a density of 3×10^6^/200 µL PBS. The cells were mixed with the same amount of Matrigel with or without exosomes and injected subcutaneously into nude mice. After 8 days, tissues were extracted, and paraffin sections were prepared to observe the tubular structure.

### Western blot analysis

Western blotting was performed according to a previous report [28]. The Rabbit anti-VEGF (19003-1-AP), Rabbit anti-FLK1 (26415-1-AP), Rabbit anti-NFDUFS4 (15849-1-AP), Rabbit anti-alpha-SYN (10842-1-AP), Rabbit anti-TH (25859-1-AP), and Rabbit anti-GAPDH (10494-1-AP) primary antibodies were purchased from Proteintech (Wuhan, China). The Rabbit anti-P38MAPK (5140), Rabbit anti-ERK1/2 (4370), Rabbit anti-p-ERK1/2 (4695), Rabbit anti-SMAD3 (9523), Rabbit anti-p-SMAD3 (C25A9), Rabbit anti-caspase3 (9662) and Rabbit anti-ICAM1(4915) primary antibodies were purchased from Cell Signaling Technology (Danvers, USA). The FITC-labeled anti-Rabbit secondary antibodies were purchased from Cell Signaling Technology (Danvers, USA).

### Immunofluorescence/immunohistochemical staining

Samples were extracted from the striatum and substantia nigra of mice, fixed in 4% paraformaldehyde at 4 °C for 0.5 h, and sent to the Servicebio company (China). The company used the same specific antibodies that we used for Western blotting.

### siRNA virus transfection

Three pairs of siRNA virus vectors targeting ICAM1 were designed and synthesized (Gene Pharma, Shanghai, China). The interference sequence was GGCTGGAG CTGTTTGAGAACA. HBMECs were transfected with the virus at a MOI of 40-50/cell using 5 mg/L polybrene (Gene Pharma, Shanghai, China), according to the manufacturer’s instructions. After transfection for 12 h, the medium was changed, and after an additional 48 h, puromycin was used to screen positive cells.

### Animal experiments

Male BALB/c mice (8-10 weeks) were obtained from the Laboratory Animal Center of the Chinese Academy of Medical Sciences (Beijing, China). The use of animals and all experimental operations were approved by the Animal Care and Use Committee of the Chinese Academy of Medical Sciences. All mice were divided into three groups. The control group received an intraperitoneal injection of PBS. The second group was injected with MPTP at a dose of 25?mg/?kg (Sigma-Aldrich, St Louis, MO, USA). The third group was injected with the same amount of MPTP as the second group and was also injected with 200 µg/mL of ESC-derived exosomes in PBS. The PD animal model was established on a five-week schedule with twice weekly injection. After the end of the five weeks, the striatum and substantia nigra were removed and fixed with 4% paraformaldehyde or frozen at -80 °C for further experiments.

### High-performance liquid chromatography (HPLC)

Frozen striatum tissue samples from the three groups were sent to the Medical Experimental Center of China Academy of Chinese Medical Sciences. The specific methods were reported in a previous study[29].

### Statistical analysis

All results were statistically analyzed using SPSS 17.0 software (IBM Corp., USA). The results were expressed as means ± standard deviation, and differences with P<0.05 were considered statistically significant.

**Figure 1.**
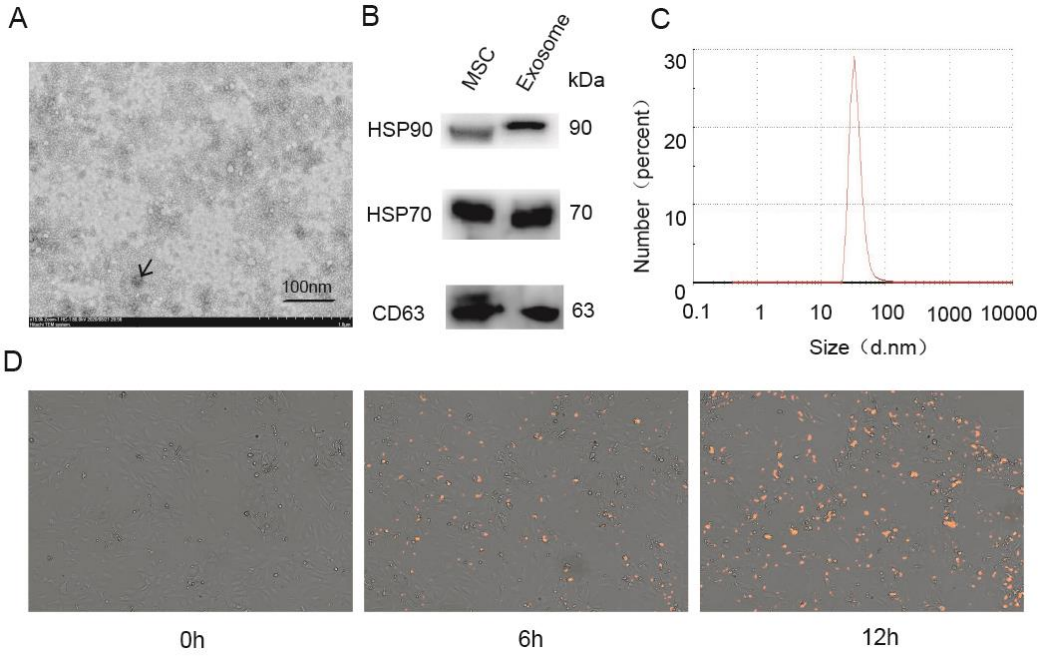
Characterization and uptake of exosomes derived from MSCs (M-Exos). (A) The morphology of M-Exos was assessed using electron microscopy. (B) The presence of HSP70, HSP90, and CD63 in M-Exos was analyzed using western blotting. (C) The size distribution of M-Exos was evaluated by NTA analysis. (D) Uptake of DiL-labeled M-Exos by HBMECs was detected at 0, 6, and 12 h.

## RESULTS

### Preparation of MSC-derived exosomes (M-Exos)

The diameter of exosomes secreted by MSCs was assessed by TEM and found to range from 30 to 200 nm (Fig. 1A). Additionally, western blot analysis was used to confirm the presence of the exosome-specific protein markers CD63, HSP70, and HSP90 (Fig. 1B). Finally, NTA analysis confirmed the approximate size range revealed by TEM, with diameters ranging from 30 nm to 100 nm (Fig. 1C). To confirm their biological activity, DiL-labeled exosomes were added into the HBMECs cultures, and real-time fluorescence microscopy after 0, 6, and 12 h showed that HBMECs absorbed large amounts of the exosomes (Fig. 1D). Overall, we found that the exosomes derived from MSCs were in the biologically active, native form, and could be used for further experiments.

**Figure 2.**
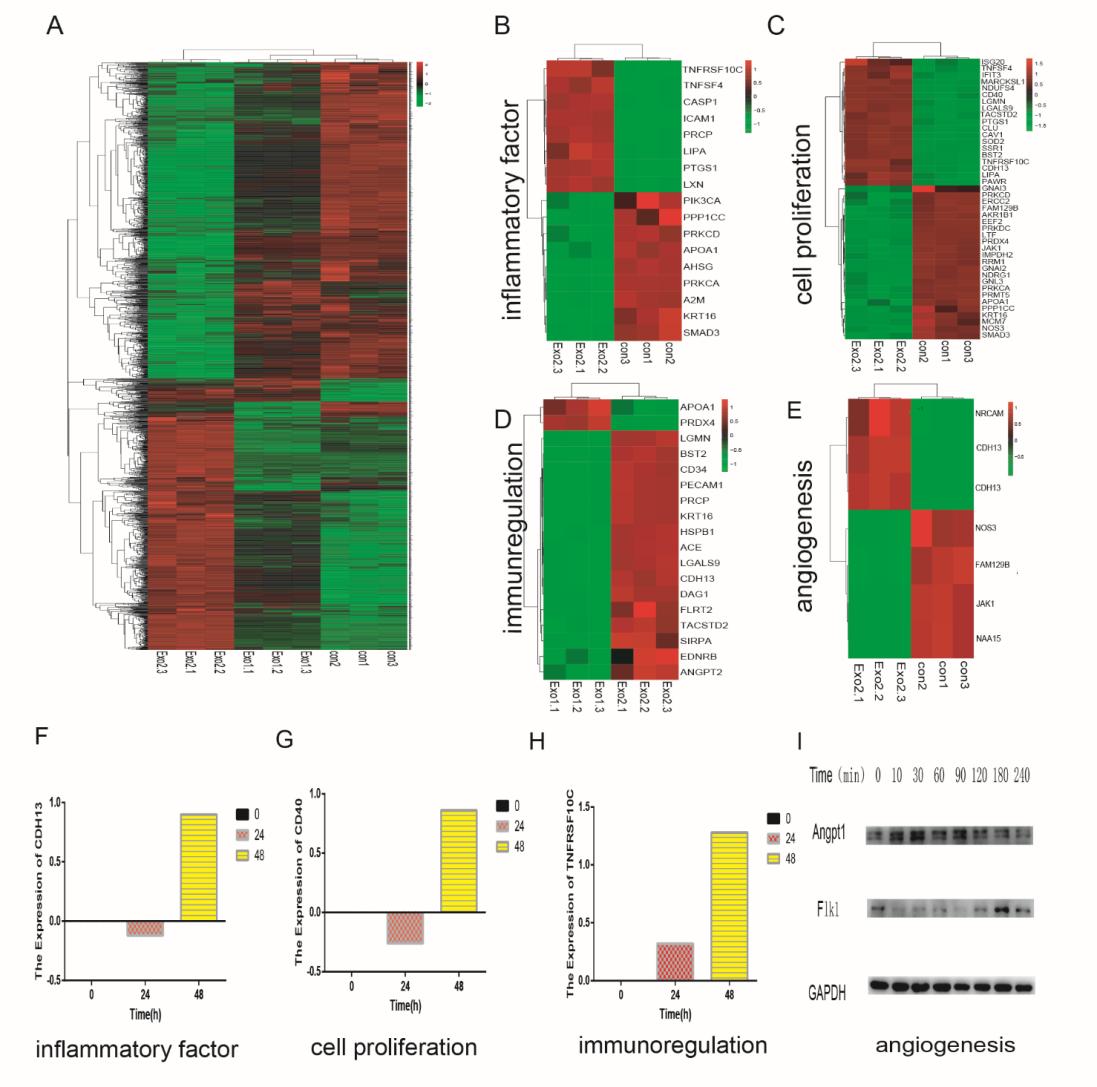
Proteomic analysis of HBMECs treated with M-Exos at different time points. (A) Heat map showing the differentially expressed genes (DEGs) in M-Exos-treated HBMECs at three different time points (0, 24, and 48 h). (B) DEGs associated with inflammatory factors. (C) DEGs associated with cell proliferation. (D) DEGs associated with immunoregulation. (E) DEGs associated with angiogenesis. (F) and (H) Detection of genes associated with inflammatory factors, cell proliferation, immunoregulation using time-series analysis. (G) Detection of genes associated with angiogenesis using western blotting (ANGPT1 and FLK1).

**Figure 3.**
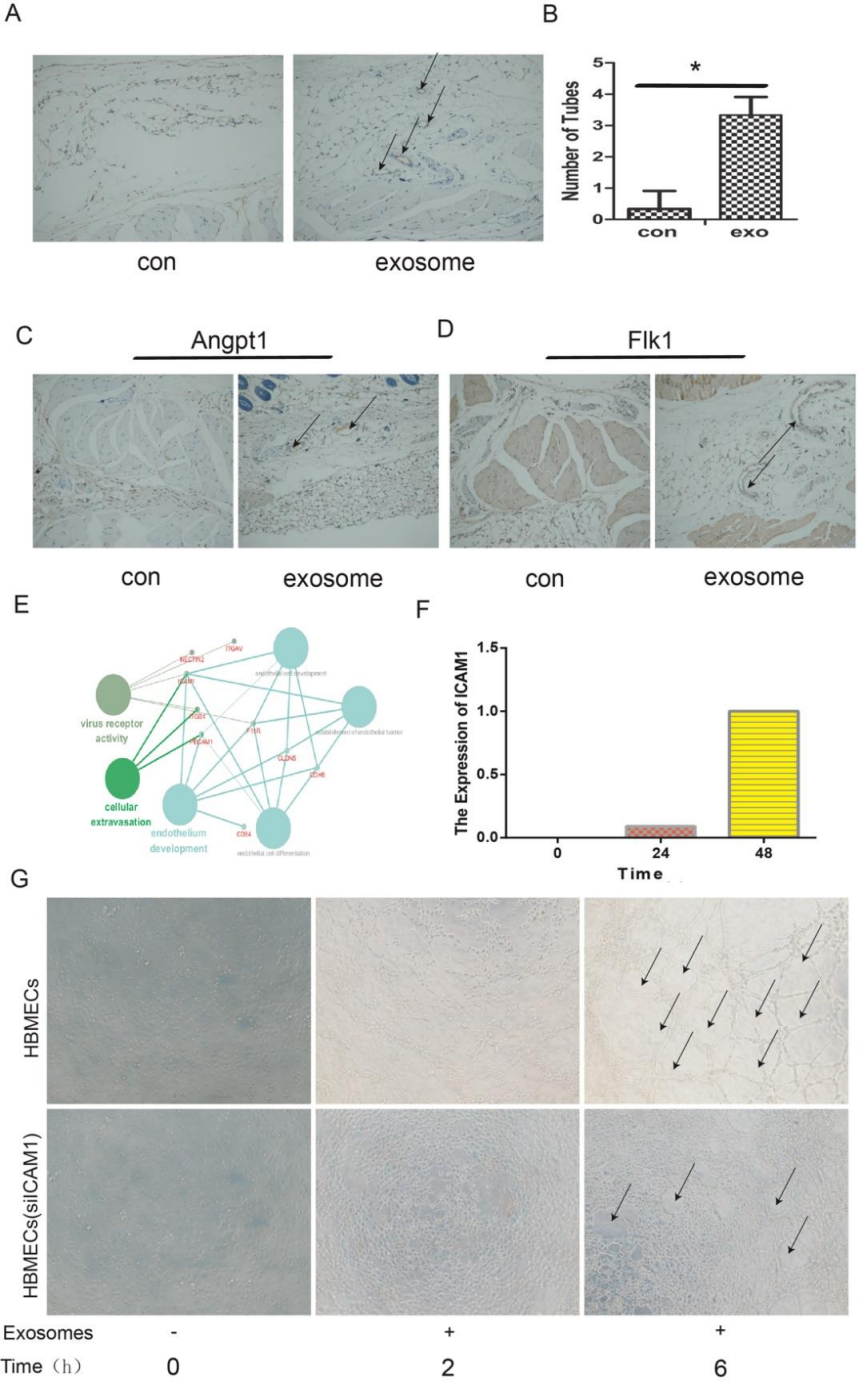
ICAM1 in HBMECs were upregulated after exosome treatment. (A) The number of tubes was detected by immune-histochemical staining (CD31). (B) The quantitative results (P<0.05). (C) Immunohistochemical staining results for the expression of ANGPT1 with control and exosomes stimulus. (D) The expression of FLK1 was detected using immunohistochemical with control and exosomes stimulus. (E-F) Proteomics and time series analysis of the target protein ICAM1, which was highly expressed after exosome stimulus. (G) The formation of tubes by HBMECs with or without ICAM1 knockdown.

### Proteomic analysis of HBMECs treated with M-Exos

We examined the alteration of genes in HBMECs treated with M-Exos by proteomic analysis. The exosomes activated the HBMECs, with increased expression of various genes at 0, 24, and 48 h (Fig. 2A). In addition, heatmap analysis revealed differently expressed functional genes, including factors related to inflammation, cell proliferation, immune regulation and angiogenesis (Figs. 2B-E). Time-series analysis indicated that the upregulation of marker genes in HBMECs is related to inflammatory factor IL3RA, proliferation-related protein CD40 and the immunoregulatory factor TNFRSF10C (Figs. 2F-H). We mainly investigated whether exosomes promoted the angiogenesis ability of the HBMECs, and therefore primarily verified the expression of angiogenesis-related genes after M-Exos treatment. The western blot results showed that the expression of the angiogenesis-related genes FlK1 and ANGPT1 was obviously enhanced (Fig. 2I). Taken together, the results confirmed that the M-Exos induced functional changes in the HBMECs.

### M-Exos regulate the angiogenesis of HBMECs by increasing the expression of ICAM1

To further investigate the impact of M-Exos on angiogenesis in vivo, we subcutaneously injected a mixture of ECM gel and HBMECs with or without 100 µg/mL of exosomes into the right forelimb of mice. Subcutaneous tissues were collected after 8 days, followed by immunohistochemical staining for CD31, which is a marker of endothelial cells. The result showed that HBMECs with exosome stimulation produced more tubular structures than the control HBMECs without stimulation (Figs. 3A/B). Moreover, immune-histochemical staining revealed that the expression of the angiogenic genes Angpt1 and Flk1 was also increased (Figs. 3C/D). Next, we assessed the secretion of ICAM1 protein, which may play a critical role in angiogenesis, by proteomics and time-series analysis (Fig. 3E). Time-series analysis showed that ICAM1 expression increasing after exosome stimulation (Fig. 3F). Moreover, when ICAM1 was knocked down in the HBMECs, the tube formation capacity was significantly reduced (Figs. 3I/J). These results suggested that M-Exos regulate angiogenesis by increasing the expression of ICAM1 in HBMECs.

**Figure 4.**
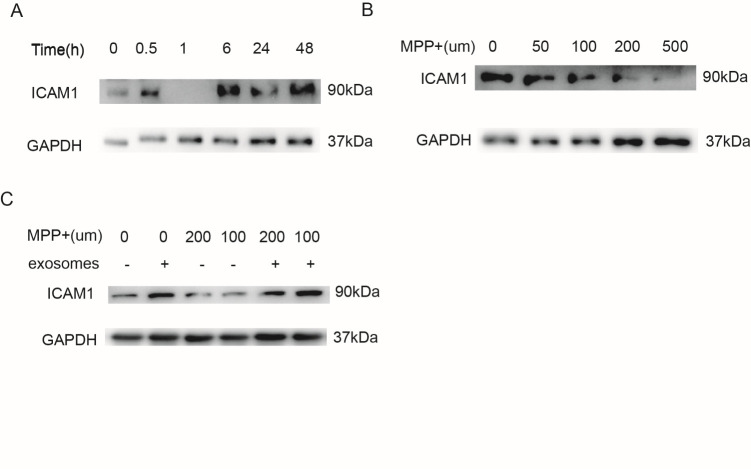
1-methyl-4-phenylpyridinium (MPP+) affects the ICAM1 expression in HBMECs. (A) The expression of ICAM1 was detected at 0, 0.5, 1, 6, 24, and 48 h after exosome treatment. (B) ICAM1 expression was negatively associated with MPP+ treatment in HBMECs. (C) M-Exos reversed the effects of MPP+ on the angiogenesis ability of HBMECs.

To further verify the possible mechanism of angiogenesis enhancement in an animal model of PD, we used 1-methyl-4-phenylpyridinium (MPP+) to establish a PD-related cell-damage model in mice. We first detected the expression of ICAM1 in the M-Exos-treated HBMECs at different time points, and the result showed that ICAM1 expression was increased by exosome stimulation in a time-dependent manner (Fig. 4A). However, when HBMECs were pretreated with MPP+ at different concentration for 48 h, the amount of ICAM1 was obviously decreased (Fig. 4B). Notably, the expression of ICAM1 was restored by stimulation with 100 µg/mL of M-Exos (Fig. 4C). Overall, these results show that exosomes derived from MSCs can promote the tube formation of HBMECs by increasing ICAM1 expression, as well as recover ICAM1 expression following MPP+ treatment.

### M-Exos promote angiogenesis via the SMAD3 and p38 MAPK signaling pathway

**Figure 5.**
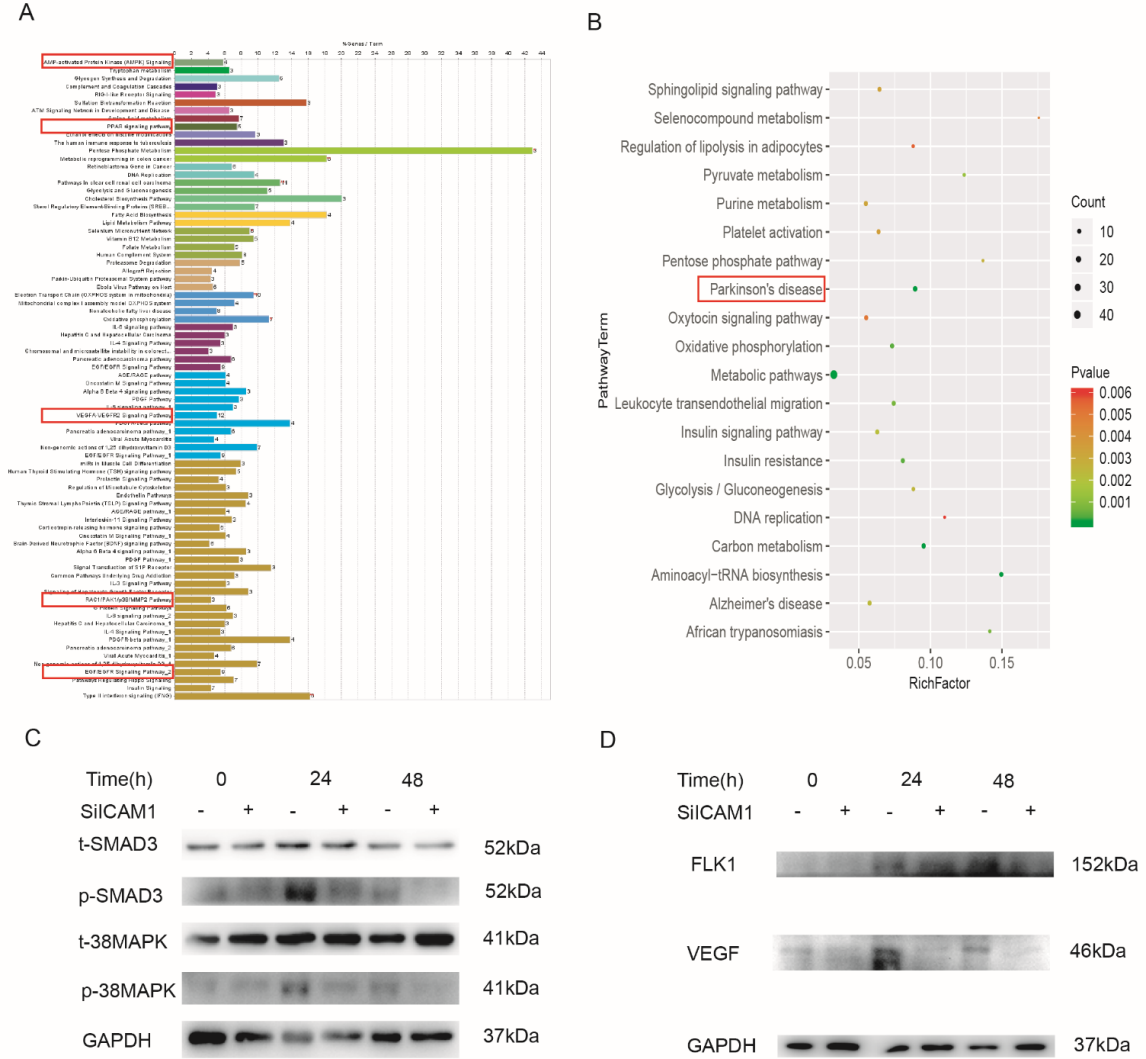
M-Exos activated the SMAD3 and p38 MAPK signaling pathways in HBMECs. (A-B) Several signaling pathways enriched in M-Exos-treated HBMECs at 48 h according to KEGG analysis. (C) Phosphorylation of SMAD3 and p38 MAPK was analyzed by western blotting in HBMECs with or without ICAM1 knockdown. GAPDH was used as the control. (D) The expression of angiogenesis-related genes (FLK1 and VEGF) in HBMECs was detected using western blotting.

To identify which signaling pathways were activated by M-Exos in HBMECs, we screened several signaling pathways in HBMECs treated with M-Exos for 48 h or mock-treated with vehicle. KEGG analysis revealed the involvement of the AMPK, p38MAPK, VEGF, PPAR, EGF and PD-related signaling pathways, as shown in Figs. 5A and B. We next confirmed the strong and rapid activation of the SMAD3 and p38 MAPK signaling pathways in HBMECs following M-Exos stimulation using western blot analysis. Moreover, the phosphorylation of factors in these two signaling pathways was significantly decreased after knocking down ICAM1 in HBMECs (Fig. 5C). In addition, the knockdown of ICAM1 in HBMECs obviously attenuated the expression of the angiogenesis-related genes VEGF and FLK1 (Fig. 5D). Therefore, the results indicated that exosomes promote the angiogenesis of HBMECs by activating the SMAD3 and p38 MAPK signaling pathways.

### M-Exos contribute to the recovery of Parkinson disease via ICAM1-mediated angiogenesis in vivo

**Figure 6.**
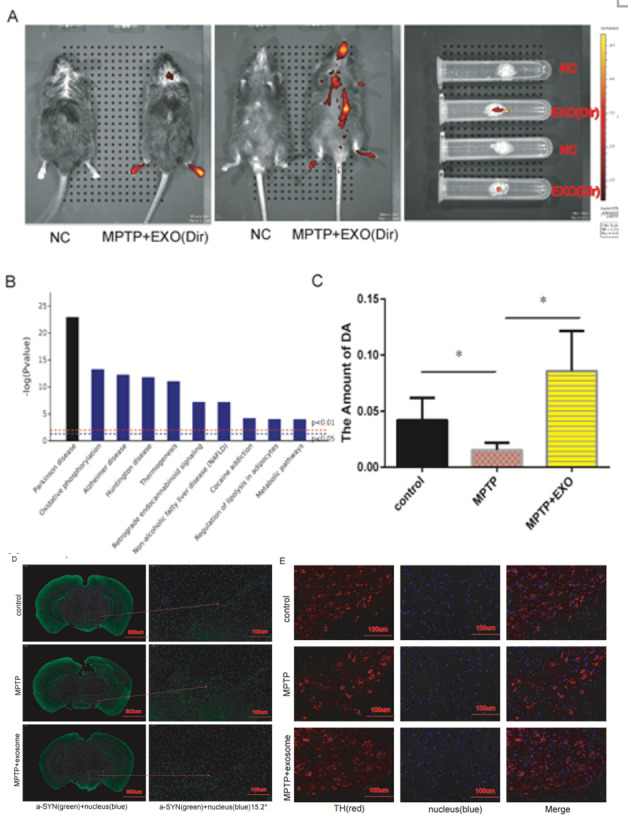
Exosomes contribute to the recovery of PD model mice in vivo. (A) DiL-labeled exosomes were intraperitoneally injected into mice, and their homing was analyzed using the Caliper IVIS Lumina II platform. (B) The differentially expressed genes in HBMECs after exosomes stimulus revealed by proteomic analysis were predicted to be associated with various diseases. (C) The amount of DA was measured in the three different groups by high-performance liquid chromatography with electrochemical detection (HPLC-ECD). (D) The expression of α-SYN in the substantia nigra region of mice from the three different groups was detected by immunofluorescence staining. (E) The number of TH-expressing positive cells in the substantia nigra region of mice from the three different groups was observed using immunofluorescence staining.

To construct a mouse model of PD, the animals were intraperitoneally injected with PBS, MPTP, or MPTP+exosomes with 10 mice in each group. When DIL-labeled exosomes intraperitoneally injected into the mice, they exhibited homing to the injured sites after MPTP treatment, including the brain and limbs (Fig. 6A). The differntially expressed proteins uncovered by proteomic analysis were found to be associated with various neurodegenerative diseases, including PD, Alzheimer's disease and Huntington’s chorea (Fig. 6B). Moreover, HPLC analysis showed that the mice in the MPTP+exosomes group had increased amounts of DA in the corpus striatum compared to the PBS and MPTP-untreated groups (Fig. 6C). To further verify the impact of exosomes on the recovery of PD in vivo, we performed immunofluorescence staining to detect cells expressing α-SYN and TH. As shown in Fig. 6D, the expression of α-SYN was significantly increased after MPTP treatment compared to the PBS group. Moreover, M-Exos clearly reversed the effects of MPTP. In addition, we observed the appearance of TH-expressing positive cells in the substantia nigra region, suggesting that the abundance of TH-expressing positive cells was decreased during the 5 weeks of MPTP treatment, while intraperitoneal injection of M-Exos could efficiently reverse this effect (Fig. 6E). Taken together, the results indicate that M-Exos can promote the recovery of PD model mice in vivo.

**Figure 7.**
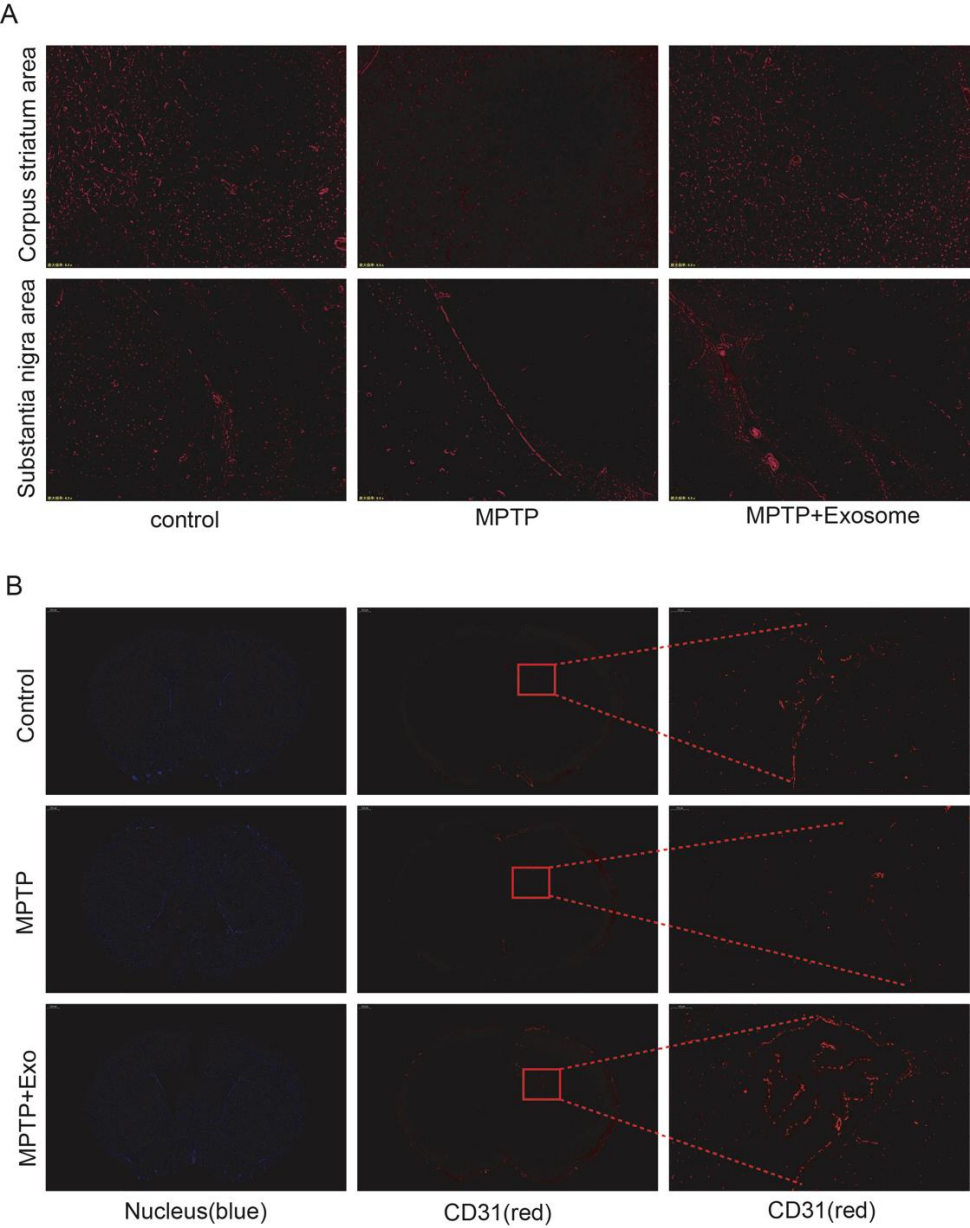
ICAM1 expression plays a significant role in the angiogenesis process in vivo. (A) Detection of ICAM1 expression in the corpus striatum and substantia nigra was carried out by immunofluorescence staining in the three different groups of mice. (B) Tube formation in the corpus striatum area was detected using immune-fluorescence staining.

It has been reported that the permeability of the blood-brain barrier (BBB) is increased in animal models of PD, and BBB dysfunction may also be associated with angiogenesis [30]. Immunofluorescence staining indicated that the expression of ICAM1 in the corpus striatum and substantia nigra of mice co-injected with MPTP and M-Exos was obviously improved compared to the MPTP group (Fig. 7A). Next, we detected the expression of the angiogenesis marker gene CD31. The results of immunofluorescence staining indicated that the expression of CD31 was obviously enhanced in the corpus striatum area after adding M-Exos compared to MPTP treatment alone (Fig. 7B). Thus, M-Exos can promote angiogenesis by increasing ICAM1 expression in a mouse model of PD, with possible therapeutic implications for human PD.

## DISCUSSION

PD is a progressive neurodegenerative disorder characterized by the large-scale loss of dopaminergic neurons in the pars compacta of substantia nigra and striatum [30]. Typical symptoms of PD include dyskinesia with resting tremors and muscle rigidity, as well as cognitive dysfunction [31]. In spite of significant research efforts, there is still no effective treatment for PD. Because PD is caused by the loss of DA neurons, their replacement is the only potentially effective therapeutic approach to fully reverse the symptoms of PD. However, long-term treatment produces various side effects, including dyskinesia [32]. Therefore, it is necessary to develop alternative approaches for the treatment of PD.

Increasing evidence indicates that MSCs are multipotent cells that can be used to treat many diseases. Exciting studies over the past years revealed that a variety of neurodegenerative diseases can be placed in remission using MSCs, including diseases that are typically fatal without effective therapies [16]. Although the specific mechanisms of the MSC-induced curative effects are poorly understood, it has been reported that they include nerve regeneration, inhibiting apoptosis, stimulating angiogenesis and immunomodulation [33]. Exosomes contain various proteins, mRNAs and microRNA [34, 35], and these components, which have been shown to promote neuronal growth and recovery [36-38], are enriched in exosomes compared with MSCs. The stimulation of angiogenesis is another positive mechanism that can contribute to the diffusion of soluble factors along newly formed blood vessels in the damaged tissue [39, 40]. In our study, we mainly elucidated how M-Exos contribute to recovery in a mouse model of PD by enhancing angiogenesis, which may provide an effective therapeutic approach for human PD.

The mechanisms through which M-Exos promote the recovery of PD are not fully understood, although reports have shown that treatment with exosomes can promote tissue-repair processes [41, 42]. The results of this study demonstrate that M-Exos can promote angiogenesis of HBMECs *in vitro* and can home to the injured sites after MPTP treatment, which promotes angiogenesis in the striatum and substantia nigra. These results suggest that M-Exos can interact with microvascular endothelial cells in the injured brain to promote angiogenesis, which is beneficial for repairing damaged blood vessels. Indeed, the present study indicates that intraperitoneal injection of M-Exos into PD model animals after MPTP treatment could decrease the aggregation of α-SYN and increase the number of TH-expressing positive cells compared to MPTP model induction without exosome treatment. At the same time, the production of DA was also obviously improved after intraperitoneal injection of M-Exos compared to the MPTP group. Beyond that, other studies have shown that M-Exos can decrease the symptoms of PD via a neuroprotective activity [22, 43].

In conclusion, ICAM1 secreted by HBMECs plays a critical role in the positive effects of M-Exos in PD model mice.
